# Heterozygosity fitness correlations and generation interval of the Norway lobster in the Aegean Sea, eastern Mediterranean

**DOI:** 10.1186/s40709-019-0103-0

**Published:** 2019-11-08

**Authors:** Georgios A. Gkafas, Marianthi Hatziioannou, Emmanouil E. Malandrakis, Costas S. Tsigenopoulos, Ioannis T. Karapanagiotidis, Elena Mente, Dimitrios Vafidis, Athanasios Exadactylos

**Affiliations:** 1 Department of Ichthyology and Aquatic Environment, School of Agricultural Sciences, University of Thessaly, Volos, Greece; 2 Institute of Marine Biology and Genetics, Hellenic Centre for Marine Research, Heraklion, Crete Greece

**Keywords:** Microsatellites, Carapace length, *Nephrops norvegicus*, Generation interval, Heterozygosity fitness correlation, Inbreeding

## Abstract

**Background:**

Comprehensively detailed information on population dynamics for benthic species is crucial since potential admixture of individuals could shift the genetic subdivision and age structure during a full breeding period. The apparent genetic impact of the potential recruitment strategy of Norway lobster *Nephrops norvegicus* is still under research. For this reason the present study was focused on genetic variation of the species over a given continuous year period in a semi-enclosed gulf of the Aegean Sea.

**Results:**

Analyses revealed that the relative smaller size class in females and the apparent faster growth of males may represent a key-role differential strategy for the two sexes, whereas females tend to mature slower. Heterozygosity fitness correlations (HFCs) showed substantially significant associations suggesting that inbreeding depression for females and outbreeding depression for males are the proximate fitness mechanisms, respectively.

**Conclusions:**

*Nephrops norvegicus* uniformal genetic composition (background of high gene flow), could be attributed to potential population recolonization, due to a hypothesized passive larval movement from deeper waters, which may suggest that some offspring of local residents and potential male non-breeders from other regions admixture randomly.

## Background

Lobsters are a quite expensive, nevertheless, valuable type of tasty seafood as they are considered as a “delicatessen” around the world. Global landings of lobsters for 2013 exceeded 230,000 mt, of which approximately 60,000 mt corresponded to Norway lobster, *Nephrops norvegicus* [1]. Pagasitikos gulf, eastern Mediterranean, is documented to be one *N. norvegicus* high population abundance site [2]. Fishing activity in Pagasitikos gulf is confined to small scale fisheries, since trawling is restricted, whilst there is a three-month period of creel ban during summer [3]. Indeed, this effective limitation of the fishing activity applied for over a decade, enhanced juvenile survival, protected stocks from overexploitation and increased yields in fishing grounds. Although overall landings of Norway lobster in Hellenic Seas, over the past 20 years were reduced by > 69% (from 1600 mt at 1989 to 490 mt at 2009) [1], fishing pressure on this species remains heavy and the species appears to be withstanding overexploitation [4].

*Nephrops norvegicus* is a marine benthic decapod crustacean (Family Nephropidae) with a wide geographical and bathymetric distribution (captured even at 400 m in northern Aegean Sea fishing grounds). It is considered as highly commercial important species resulting in a recent interest as a new candidate species for aquaculture [3, 5, 6]. A high larval dispersal ability, although dependent on sea currents, such as many marine organisms [7], has been recorded also for *N. norvegicus* [8].

Knowledge of its population dynamic pattern (a background of quite high gene flow was previously recorded [9, 10]) will elucidate how genetic variation is partitioned among populations, thus, having important implications not only in Norway lobster’s evolutionary biology and ecology, but also in implementing conservation biology strategies. However, the current understanding on *N. norvegicus* ecology and evolution was focused so far on classical ecological [2, 11–13] and reproductive [3] approaches with, nevertheless, valuable information on the molecular level [9, 14–17]. Multiple evolutionary processes can affect the temporal and spatial variability of allelic frequencies in natural populations (e.g. [18]); such are migration, mutation, selection and genetic drift. Although marine species have the potential of migrating through long distances, genetic markers could reveal the presence of small, or large scale genetic structure (e.g. [19]). Since Norway lobster exhibits a burrowing lifestyle as an adult, the recorded lack of significant genetic differentiation could be mostly attributed to population mixing during larval pelagic phase. Indeed, *N. norvegicus* larval stage exceeds 50 days in plankton, before benthic settlement occurs. Juveniles appear to preferentially take up residence in vacated adult burrows, otherwise constructing their own burrows as an extension of the already existing ones [20]. Notwithstanding, larvae are able to migrate 100–300 km depending on local oceanographic characteristics and water mass transportations [21]. Interestingly, low genetic variation was recorded through the species distribution, mainly due to genetic drift [22]. However, human activities could potentially modify the environment of larvae dispersal such as sound and light pollution, shipping and most notably pollution crisis (e.g. oil spills).

It has been documented that during the protracted brooding periods and the periods of extended release of eggs, alteration of age and sex ratios in Norway lobster is possible [3]. Thus, such periods may influence population dynamics, in the sense of genetic differentiation, or even in subsequent recruitment of the species. On the other hand, this potential admixture of individuals could shift temporally the genetic subdivision and size structure during a full breeding period. However, the apparent genetic impact of the potential recruitment strategy is still under investigation. Consequently, by measuring the genetic diversity, it may be possible to assess the mechanism that generates a potential correlation between heterozygosity and life-history traits of body size of the species. The morphology of Norway lobster has already been thoroughly described [3] and it is well documented that growth play an important role to life-history success of the species in terms of reproduction. Published data have shown that heterozygosity is often correlated with indirect fitness measurements such as fluctuating asymmetry [23–26] and length measurements [27, 28]. According to the theory, low heterozygous individual have a relative reduced fitness, possibly due to inbreeding depression. Many earlier studies used microsatellite DNA markers, and due to the nature of these markers, they are not considered to represent genome-wide variability [29]. However, most important is not the panel of the markers used, but the level of identity disequilibrium in the studied populations [30]. On the other hand, more recent studies demonstrated the greater power availed by genome sampling (High-throughput sequencing, e.g. [31]), revealing new insights in genetic variability, which is however subjected to costs and high performance computing analyses.

To test this hypothesis, the present study was focused on assessing genetic variation in *N. norvegicus* over a continuous year period. Also, the generation interval of the given year was calculated in order to assess the age overlap of the species in Pagasitikos gulf. For this reason microsatellite markers were used as a molecular genetic tool and proved to be a comprehensively informative approach in the study area. Morphometric data were combined with the allele frequencies of *N. norvegicus* at a temporal scale in order to assess the generation interval separately for the two sexes. Moreover, heterozygosity-fitness correlations were tested regarding levels of genetic diversity and carapace length variability.

## Methods

### Sampling

**Fig. 1:**
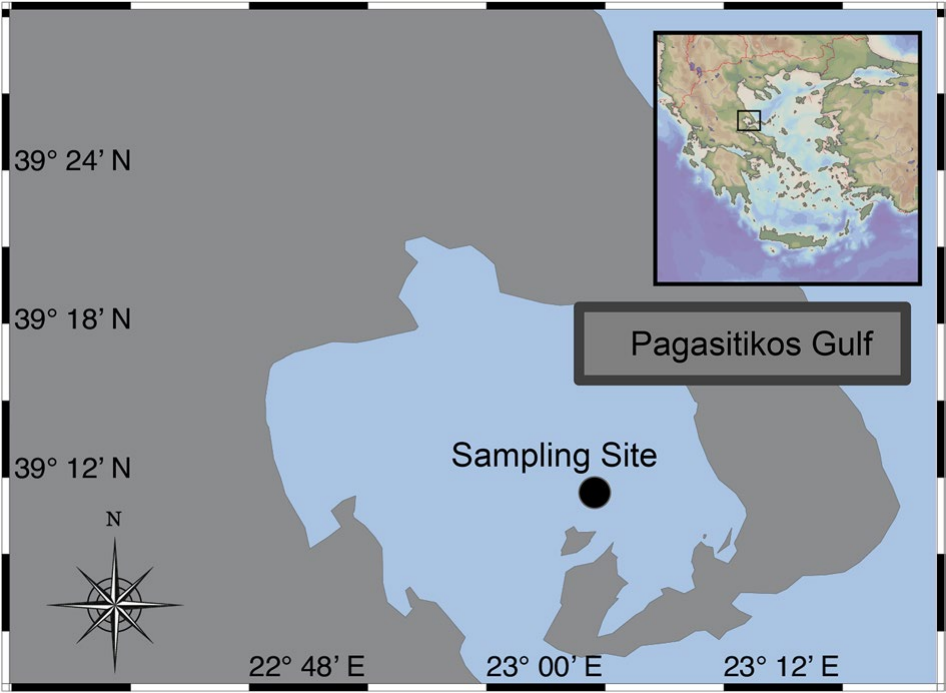
Study area of Pagasitikos gulf and sampling site (black dot) of the experimental trawling (after [2])

A sampling scheme was designed in order to survey temporal variation of Norway lobster species during a full breeding period. Sampling of male and female individuals of *Nephrops norvegicus* was carried out during a given continuous year (2007) near the deepest area of Pagasitikos gulf (39°16′N; 23°02′Ε) (Fig. 1). It is worth mentioning that Pagasitikos gulf was selected because it is considered a sampling area of high *N. norvegicus* abundance throughout the Aegean Sea [13]. A total number of 764 specimens were collected through experimental trawling from a registered fishing ground approximately in the middle of a given month (subject to availability) (Additional file 1: Table S1). Following individual weighting and measuring, white muscle was dissected and stored at − 20 °C. Carapace length was measured on each specimen; measurements were taken to the nearest 0.1 mm.

### DNA extraction and amplification strategies

DNA was extracted from 50 mg of white muscle tissue from each individual using the standard phenol–chloroform protocol [32]. DNA pellet was finally diluted in 50 μL TE (10 mM Tris–HCl, 1 mM EDTA, pH 8.00) and stored at − 20 °C, for downstream PCR. Quantity and quality of template DNA were confirmed by measuring absorbance at 260 nm and 260/280 ratio, respectively, using a Nanodrop 1000 spectrophotometer (Thermo Fisher Scientific, Wilmington, USA).

Six microsatellite loci, specific to Nephropidae family [14, 33], were tested based on their recorded polymorphism in order to produce scorable amplification patterns (Table 1). Amplification reactions were carried out in a 20 μL volume using 10 ng of extracted genomic DNA, 2 mL reaction buffer (10×), 0.25 mM of each dNTP, 3 mM MgCl_2_, 15 μM of each primer, 1 U *Taq* polymerase (KAPABIOSYSTEMS, Massachusetts, USA), and ddH_2_O, up to the final volume. For all reactions, cycling conditions were 94 °C for 30 s, 57 °C for 30 s, followed by 72 °C for 30 s. Microsatellites were amplified with fluorescently labeled forward primers and PCR products were run with the internal ladder Genescan-500 LIZ Size Standard (Applied Biosystems, Foster City, CA, USA) in an ABI3700 Automated Sequencer (Applied Biosystems, Foster City, CA, USA). Genotypic data were analyzed using the STRAND 2.3.0.48 software package [34].

**Table 1:** Gene diversity (G), number of alleles (N_o_), allelic richness (R), inbreeding coefficient index (F_IS_) per locus and sex for *N. norvegicus*

Loci	Females				Males			
	G	N_o_	R	F_IS_	G	N_o_	R	F_IS_
*Nnmic2*-*E4*	0.898	15	14.158	*0.157*	0.898	10	9.976	− 0.054
*Nnmic1*-*F2*	0.955	23	22.168	*0.122*	0.941	21	20.232	0.024
*Nnmic1B11*	0.830	8	7.834	*0.153*	0.825	10	9.644	*0.247*
*Nnmic1*-*C12*	0.716	9	8.129	− 0.084	0.676	7	6.765	− 0.438
*NnmicT*-*G2*	0.958	24	23.068	*0.125*	0.941	23	21.845	− 0.033
*Lobp3*	0.797	11	10.620	*0.186*	0.783	11	10.485	0.034
*Total*	0.859	15	12.559	*0.109*	0.844	13.66	13.157	− 0.041

Females			Males					
MLH	IR	HL	MLH	IR	HL			
0.528 ± 0.23	0.471 ± 0.23	0.467 ± 0.23	0.533 ± 0.15	0.456 ± 0.156	0.464 ± 0.16			

	CI 95%	t-value	DF	*p-*value				
*MLH*	0.0057 (− 0.0638; 0.0752)	0.16	88	0.872				
*IR*	− 0.0057 (− 0.0754; 0.0640)	− 0.16	88	0.872				
*HL*	− 0.0029 (− 0.0745; 0.0688)	− 0.08	85	0.937				

Mean values of MLH, IR and HL for both sexes along with confidence intervals (CI) values of *t* test; significances are in italics

### Data analysis

All loci were tested for the presence of null alleles, or allelic dropout using the software MICROCHECKER v.2.2.3 [35]. The software Bayescan v.1.0 [36] was used to identify candidate loci under natural selection. Exact tests for Hardy–Weinberg equilibrium and Linkage Disequilibrium (using Fisher’s exact tests) were carried out using the software Genepop v.1.2 [37]. F_IS_ index [38], number of alleles, allelic richness and gene diversity per locus and per sex were calculated using the FSTAT v.2.9.3.2 software [39].

Species generation interval was assessed with the AgeStructure software [40] based on genotype parentage assignment. Length Frequency Distributions (LFD) were calculated separately for male and female individuals per month using carapace length. Class interval was calculated as 3.72 mm when using the formula described by Sokal and Rohlf [41]. All LFD analyses were carried out in SPSS 14.0 (SPPS Inc., Chicago, USA).

Mean multilocus heterozygosity (MLH) and inbreeding measures (Internal Relatedness-IR and Homozygosity by Locus-HL) were performed using the software IRmacroN v.4.0, an EXCEL macro written in Visual Basic [42]. Linear regressions were used to investigate possible relationships between measures of genetic diversity and carapace length for both sexes using Minitab v.17.0 (Minitab Ltd., Coventry, UK).

## Results

Factorial Correspondence Analysis (FCA) reveal a single population (Additional file 1: Fig. S1), thus individuals were treated as such. Significant departures from Hardy–Weinberg equilibrium occurred at random loci and Linkage Disequilibrium was found at multiple loci pairs. On the other hand, no evidence of selection was detected across all six loci. Female individuals favored significant evidence of high inbreeding index (overall F_IS_ = 0.109, *p *< 0.05), while in male individuals this was not the case (overall F_IS_ = − 0.041; *p *> 0.05). The number of alleles ranged from 9 (*Nnmic1*-*C12*) to 24 (*NnmicT*-*G2*) with a mean value of MLH at 0.528 and 0.533 for female and male individuals, respectively. Summary statistics on genetic variability are presented in Table 1.

**Fig. 2:**
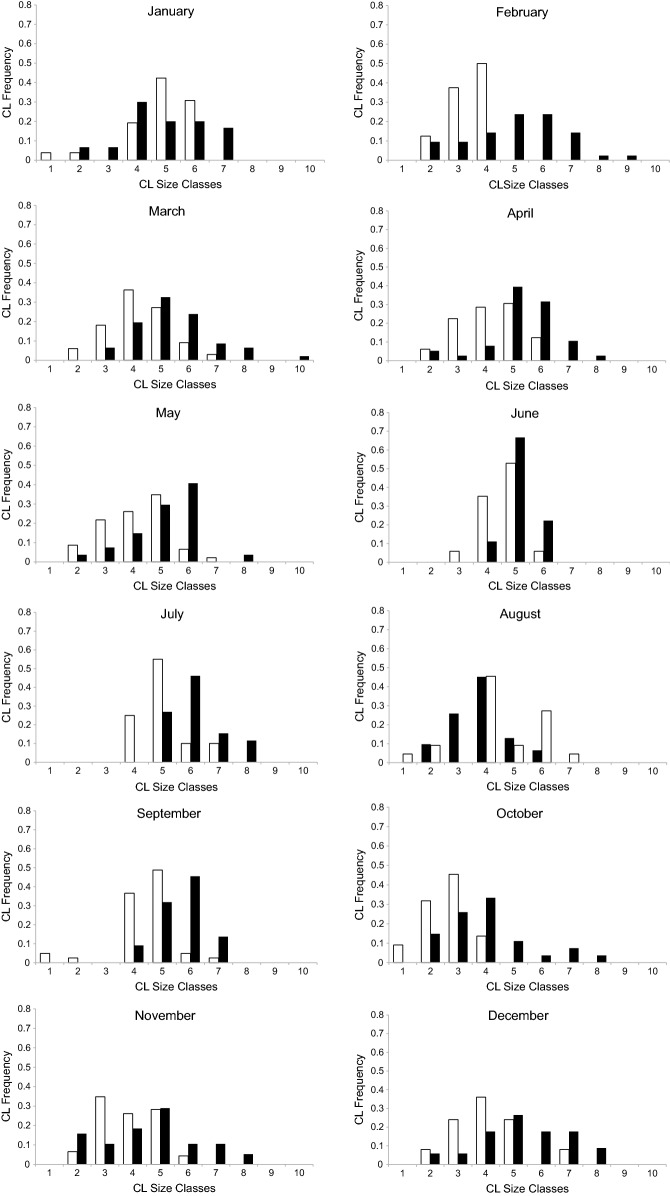
Frequencies of Carapace Length (CL) distribution in each size class per month, separately for female (white bars) and male (black bars) individuals

**Fig. 3:**
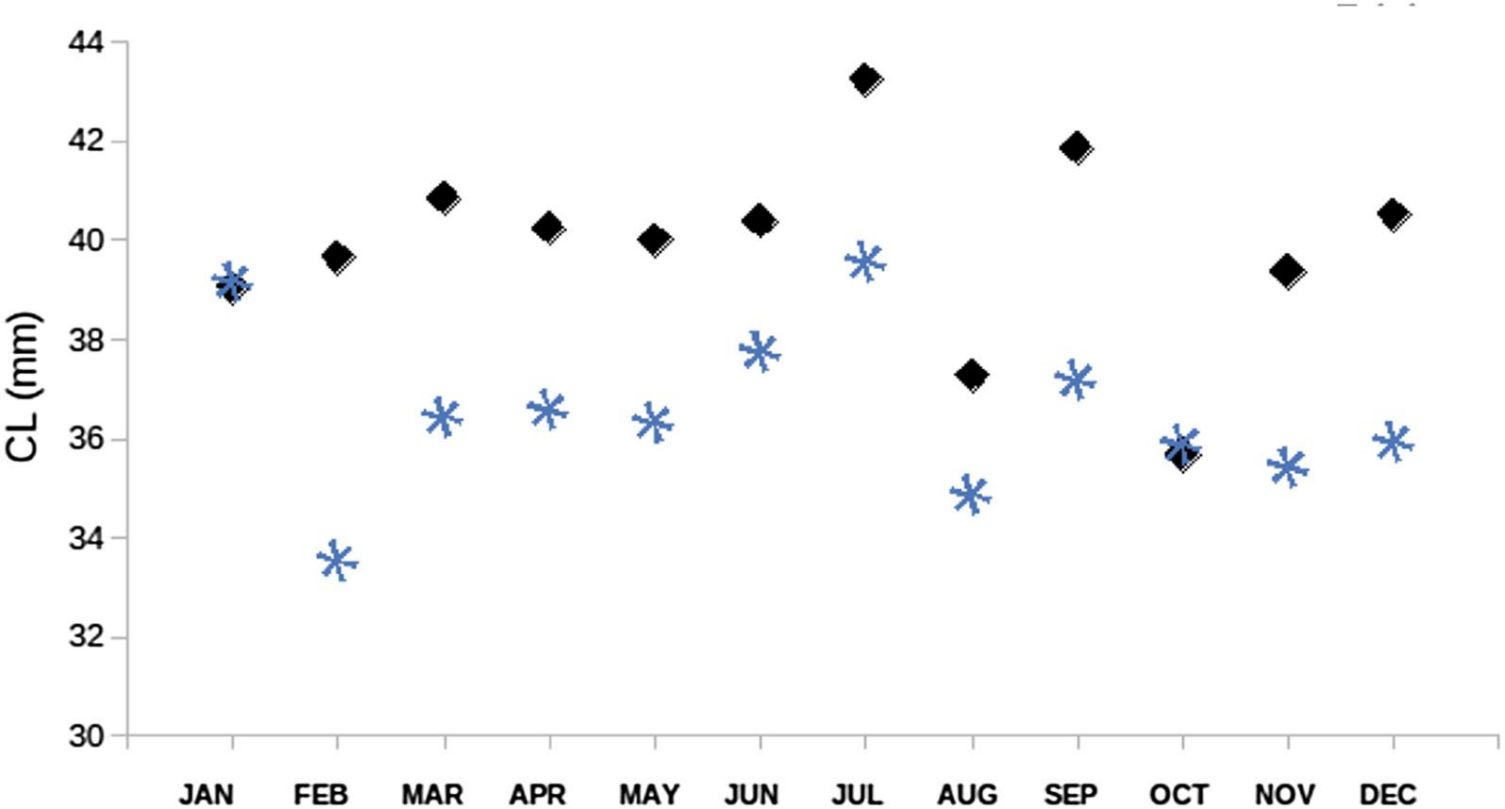
Plot of mean Carapace Length per month for each sex. Asterisks denote females and diamond-shape males, respectively

Mean Generation interval was calculated at 5.41 years (CI 95% 5.25–5.71), which is considered rather informative (Table 2), since previous ecological data remain unclear on age clustering of the species [6]. Generation interval for female and male individuals was calculated at 6.28 and 5.89, respectively. Each individual was assigned accordingly to each carapace length class (see review in [43]) as shown in Fig. 2. Female lobsters attain sexual maturity at approximately 2.5–3 years of age at a carapace length of 21–22 mm. Males become mature after 3 years at a carapace length of 25 mm [44]. The present dataset recorded a female size at the onset of maturity at 28.1 mm [3], a fact which is consistent with the proposed generation interval calculation. Monthly pairwise differentiation of mean carapace length per sex was significant for all comparisons (Chi square analysis), except for January and October (Fig. 3).

**Table 2:** Estimates of generation effective size (N_e_) and generation interval (GI) for *N. norvegicus* sampled individuals

	N_e_	GI paternal	GI maternal	Overall GI
Estimate	1902	5.89	6.28	5.41
CI 95% low	1749	5.89	6.08	5.25
CI 95% upper	2444	5.97	6.69	5.71

**Fig. 4:**
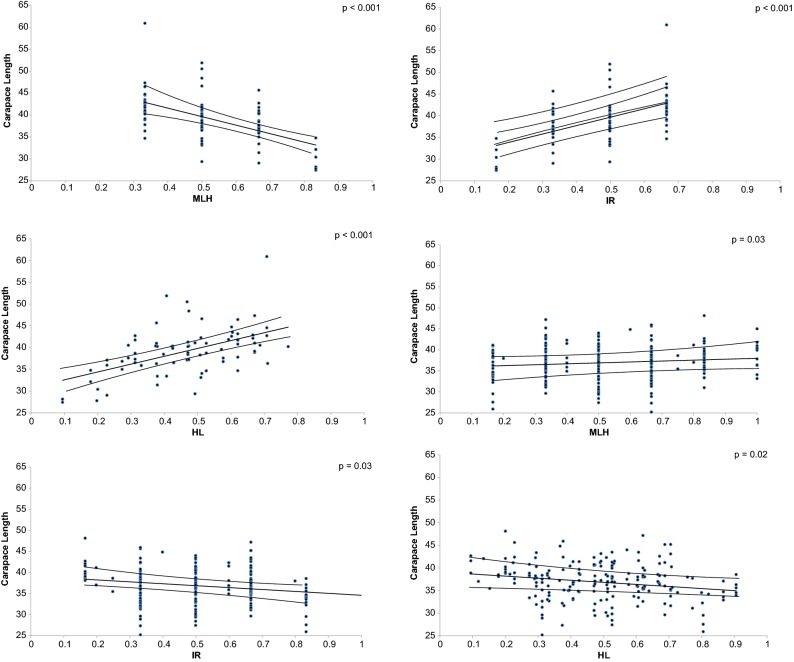
Regression plots of the three different measures of heterozygosity against the carapace length in male individuals (**a** MLH; **b** IR; **c** HL), and female individuals (**d** MLH; **e** IR; **f** HL). Dashed lines represent confidence intervals (95%)

MLH and inbreeding measures (IR, HL) did not differ significantly between male and female individuals (Table 1). On the contrary, HFC analysis showed significant associations for all three different measures of heterozygosity against the carapace length for both male and female individuals (Fig. 4). Interestingly, male individuals showed significant negative correlation with respect to MLH, IR and HL, suggesting outbreeding depression. The correlations in male individuals was strongly negative regarding the carapace length measure (Table 3) and remained highly significant even after Bonferroni correction (*r*_*MLH*_^2^ = 0.352, *p *< 0.001; *r*_*IR*_^2^ = 0.352, *p *< 0.05; *r*_*HL*_^2^ = 0.306, *p *< 0.001). On the other hand, female individuals showed relatively low, but significantly positive association (*r*_*MLH*_^2^ = 0.047, *p* = 0.03; *r*_*IR*_^2^ = 0.047, *p* = 0.03; *r*_*HL*_^2^ = 0.052, *p* = 0.023) with carapace length (Table 3), implying that inbreeding depression, this time, is the main fitness mechanism.

**Table 3:** Regression analysis of variance of carapace length against MLH, IR and HL for female and male *N. norvegicus* sampled individuals; significances are in italics

	MLH	IR	HL
Females			
Equation	y = 33.30 + 4.053x	y = 37.34 − 4.042x	y = 37.39 − 4.188x
*r*^2^	0.047	0.047	0.052
*F*	4.870	4.870	5.36
*p* value	*0.030*	*0.030*	*0.023*
Males			
Equation	y = 54.01 − 24.89x	y = 29.18 + 24.83x	y = 30.42 + 22.22x
*r*^2^	0.352	0.352	0.306
*F*	17.95	17.95	14.55
*p* value	*0.001*	*0.001*	*0.001*

## Discussion

This study represents one of the first attempts dealing with the ecological aspects of mixed gene pools in the marine environment, regarding different reproductive strategies with respect to sex. Male and female Norway lobsters, surprisingly, favored different evolutionary mechanisms suggesting that even relatively high migration movements (i.e. outbreeding), or high inbreeding could adapt robustly in terms of fitness. Previous genetic studies in *Nephrops norvegicus* demonstrated a low but significant genetic heterogeneity [9, 16] through the species distribution. However, an unclear geographical pattern among lobster populations has been recorded; an IBD model of geographical and genetic distances was not valid [9]. These findings have been discussed under a background of high gene flow, thus temporal sampling over differential generations was of great interest among *N. norvegicus* populations, in order not only to test if the observed genetic pattern remains stable over time, but also in order to clarify the model of genetic and size classes structuring.

In both sexes we observed significant but different HFC regression slopes according to carapace length. In males, the associations of different levels of heterozygosity with the fitness related trait were negative, suggesting that outbreeding depression is likely to be the substantial conservation scenario due to an apparent movement of lobsters. The latter suggests that, highly heterozygous male individuals grew less and suffered much greater loss of fitness. The mechanism that underlies this observed pattern may be local adaptation; offspring of genetically distant mates may be less adapted to the environment than their parents [45]. In relatively small sized populations of several other species, local adaptation is often attributed to impose a strong impact on HFC and therefore reflects outbreeding depression [26, 46–48].

Despite this observed alteration in allele frequencies, little variation in levels of multilocus heterozygosity was recorded, suggesting high levels of inbreeding in female individuals. Interestingly, female individuals showed a rather low but also significant relationship between carapace length and overall heterozygosity measures, implying that inbreeding depression this time, is the primary mechanism for such an association. Indeed, high levels of F_IS_ may suggest non-random mating, thus indicating high inbreeding within females. Given this pattern, the observed loss of allelic frequencies from the apparent local population, might imply some degree of adaptiveness to the local environment. The specific analyzed microsatellite loci were somewhat variable in all females and most of the individuals were homozygous at all these six loci. Such a documented absence of a fine-scale structure of the species to date [9, 16], may lead to smaller local effective population sizes, and the possibility of a greater impact of inbreeding on fitness [49].

Moreover, significant levels of HFC differentiation among the two sexes could be explained by the actual migration of individuals favoring rather low relatedness within the population, suggesting a putative replacement by immigrants. To this extent, this study illustrates that the near-panmictic *N. norvegicus* populations may profound a temporal genetic variation in a local-scale level, demonstrating the presence of a non-inbreeding enhancement as a process of rare alleles transition. Although high inbreeding levels within temporal samples could affect the effective population size promoting low genetic differentiation among samples, the presence of non-breeders seems to have a greater local genetic impact than the dispersal of in-breeders. Nonetheless, the level of gene diversity was moderately high, implying either a degree of structuring, or an exchange of genes occurred in the past. Thus, taking into account the presently calculated mean generation interval of approximately 5.41 years for *N. norvegicus*, one could assume that at least one effective migrant each year might explain the recorded levels of pairwise genetic differentiation. It has been suggested [50] that a minimum of one and a maximum of ten migrants per generation would be the appropriate empirical rule for genetic conservation purposes.

The differential status with regards to sex was also profound in size classes. Pairwise comparisons of the mean length-at-size class showed that female individuals were smaller compared to males. This may be due to the different reproductive behavior of the species in question, resulting to decreased catchability of female individuals [51]. On the other hand, due to the continuous breeding period [3] females’ adaptation possibly rests to their reproduction strategy rather than growth, thus resulting in lower growth rates [12, 41, 51]. The documented relative smaller size classes in females and the faster growth of males maybe represent a key-role differential strategy for the two sexes, whereas females tend to mature slower. Indeed, paternal generation interval is smaller compared to the maternal one, indicating a cryptic and complex social behavior. Nevertheless, the apparent movement of male individuals as stated in the genetic analyses might explain at some extent the differences in size classes between the two sexes. The differences in monthly mean carapace length between the two sexes were found to be statistically significant in most of the cases. Non-significance in January and October might be explained by the fact that the brooding period presented the highest peaks just before these 2 months [3].

Sea water circulation pattern of the Aegean Sea depicts a far eastern movement of surface currents along Chalkidiki Peninsula (see [52]), following the eastern Greek mainland coastline entering Pagasitikos gulf through the Trikeri Strait with several, throughout, inflow and outflow patterns (see [53]). Evoikos gulf communicates with the Aegean Sea through Oreoi Channel and is mainly associated with frequent and intense tidal water movements (Fig. 1). The overall oceanographic pattern facilitating larval movements through these areas for several marine species amplifies the precautions that should be taken into account, when recruitment and gene pool conservation strategies are implemented for *N. norvegicus*, besides specificities on fishing, spawning and feeding grounds/banks [54].

## Conclusions

Conclusively, the northern/central Aegean Sea is subjected to a strong influence of more eutrophic waters compared to the southern Aegean, featuring higher zooplankton abundance. Richness of a number of benthic species was negatively correlated with depth, partly reflecting the intense research activities in shallower waters and the poor scientific knowledge of the deeper ones [55]. In that sense, *N. norvegicus* uniformal genetic composition (background of high gene flow), could be attributed to potential population recolonization, due to a hypothesized passive larval movement from deeper waters, which may suggest that some offspring of local residents and potential male non-breeders from other regions admixture randomly. Norway lobster has relatively high F_IS_ values in the study area, suggesting that potential populations of the central Aegean Sea need to be identified and to apply conservation measures. Considering the above along with the apparent absence of physical barriers, individuals of the species in question within the study area may be favored to recruitment from an apparent nearby large population encountered in deeper waters as local fishermen claim.

## Supplementary information

**Additional file 1: Table S1.** Number of individuals per gender and month. **Figure S1.** Factorial Correspondence Analyses for all individuals. Result suggest a single population.

## Data Availability

The data have been presented with the article.

## References

[1] FAO. 2018. The State of World Fisheries and Aquaculture 2018—Meeting the sustainable development goals. Rome. Licence: CC BY-NC-SA 3.0 IGO. 2018. p. 44.

[2] Smith CJ, Papadopoulou KN (2003). Burrow density and stock size fluctuations of *Nephrops norvegicus* in a semi-enclosed bay. ICES J Mar Sci.

[3] Mente E, Karapanagiotidis IT, Logothetis P, Vafidis D, Malandrakis E, Neofitou N (2009). The reproductive cycle of Norway lobster. J Zool.

[4] Sardá F (1998). *Nephrops norvegicus* (L.): comparative biology and fishery in the Mediterranean Sea. Introduction, conclusions and recommendations. Sci Mar..

[5] Rosa R, Nunes ML (2003). Biochemical composition of deep-sea decapod crustaceans with two different benthic life strategies off the Portuguese south coast. Deep-Sea Res I.

[6] Smith C, Papadopoulou KN, Mente E (2008). The crustacean *Nephrops norvegicus*: growth and reproductive behaviour. Reproductive biology of crustaceans: case studies of decapod crustaceans.

[7] Exadactylos A, Mente E (2008). Aspects on population and aquaculture genetics of crustaceans. Reproductive biology of crustaceans: case studies of decapod crustaceans.

[8] Hill AE, White RG (1990). The dynamics of Norway Lobster (*Nephrops norvegicus* L.) populations on isolated mud patches. ICES J Mar Sci..

[9] Stamatis C, Triantafyllidis A, Moutou KA, Mamuris Z (2004). Mitochondrial DNA variation in northeast Atlantic and Mediterranean populations of Norway lobster, *Nephrops norvegicus*. Mol Ecol..

[10] Pampoulie C, Skirnisdottir S, Hauksdottir S, Olafsson K, Eiríksson H, Chosson V (2011). A pilot genetic study reveals the absence of spatial genetic structure in Norway lobster (*Nephrops norvegicus*) on fishing grounds in Icelandic waters. ICES J Mar Sci.

[11] Chapman CJ, Rice AL (1971). Some direct observations on the ecology and behaviour of the Norway lobster *Nephrops norvegicus*. Mar Biol.

[12] Tuck ID, Chapman CJ, Atkinson RJA, Bailey N, Smith RSM (1997). A comparison of methods for stock assessment of the Norway lobster, *Nephrops norvegicus*, in the Firth of Clyde. Fish Res.

[13] Smith CJ, Marrs SJ, Atkinson RJA, Papadopoulou KN, Hills JM (2003). Underwater television for fisheries-independent stock assessment of *Nephrops norvegicus* from the Aegean (eastern Mediterranean) Sea. Mar Ecol Prog Ser.

[14] Streiff R, Guillemaud T, Alberto F, Magalhães J, Castro M, Cancela ML (2001). Isolation and characterization of microsatellite loci in the Norway lobster (*Nephrops norvegicus*). Mol Ecol Notes.

[15] Streiff R, Mira S, Castro M, Cancela ML (2004). Multiple paternity in Norway lobster (*Nephrops norvegicus* L.) assessed with microsatellite markers. Mar Biotechnol..

[16] Stamatis C, Triantafyllidis A, Moutou KA, Mamuris Z (2006). Allozymic variation in Northeast Atlantic and Mediterranean populations of Norway lobster, *Nephrops norvegicus*. ICES J Mar Sci..

[17] Aurelle D, Baker AJ, Bottin L, Brouat C, Caccone A, Chaix A (2010). Permanent genetic resources added to the molecular ecology resources database 1 February 2010-31 March 2010. Mol Ecol Resour..

[18] Wright S (1951). The genetical structure of population. Ann Eugenic..

[19] André C, Larsson LC, Laikre L, Bekkevold D, Brigham J, Carvalho GR (2011). Detecting population structure in a high gene-flow species, Atlantic herring (*Clupea harengus*): direct, simultaneous evaluation of neutral vs putatively selected loci. Heredity.

[20] Tuck ID, Atkinson RJA, Chapman CJ (1994). The structure and seasonal variability in the spatial distribution of *Nephrops norvegicus* burrows. Ophelia..

[21] Marta-Almeida M, Dubert J, Peliz A, Dos Santos A, Queiroga H (2008). A modelling study of Norway lobster (*Nephrops norvegicus*) larval dispersal in southern Portugal: predictions of larval wastage and self-recruitment in the Algarve stock. Can J Fish Aquat Sci.

[22] Maltagliati F, Camilli L, Biagi F, Abbiati M (1998). Genetic structure of Norway lobster, *Nephrops norvegicus* (L.) (Crustacea: Nephropidae), from the Mediterranean Sea. Sci Mar..

[23] van Valen L (1965). A study of fluctuating asymmetry. Evolution.

[24] Hoelzel AR, Fleischer RC, Campagna C, Le Boeuf BJ, Alvord G (2002). Impact of a population bottleneck on symmetry and genetic diversity in the northern elephant seal. J Evol Biol.

[25] Neff BD (2004). Stabilizing selection on genomic divergence in a wild fish population. Proc Natl Acad Sci USA.

[26] Borrell YJ, Pineda H, McCarthy I, Vázquez E, Sánchez JA, Lizana GB (2004). Correlations between fitness and heterozygosity at allozyme and microsatellite loci in the Atlantic salmon, *Salmo salar* L. Heredity..

[27] Lesbarrères D, Pagano A, Lodé T (2003). Inbreeding and road effect zone in a Ranidae: the case of Agile frog, *Rana dalmatina* Bonaparte, 1840. C R Biol..

[28] Hoffman JI, Hanson N, Forcada J, Trathan PN, Amos W (2010). Getting long in the tooth: a strong positive correlation between canine size and heterozygosity in antarctic Fur Seals *Arctocephalus gazella*. J Hered.

[29] Miller JM, Coltman DW (2014). Assessment of identity disequilibrium and its relation to empirical heterozygosity fitness correlations: a meta-analysis. Mol Ecol.

[30] Balloux F, Amos W, Coulson T (2004). Does heterozygosity estimate inbreeding in real populations?. Mol Ecol.

[31] Hoffman JI, Simpson F, David P, Rijks JM, Kuiken T, Thorne MAS (2014). High-throughput sequencing reveals inbreeding depression in a natural population. Proc Natl Acad Sci USA.

[32] Sambrook J, Fritsch EF, Maniatis T (1989). Molecular cloning: a laboratory manual.

[33] Hodgins-Davis A, Roberts S, Cowan DF, Atema J, Bennie M, Avolio C (2007). Characterization of SSRs from the American lobster, *Homarus americanus*. Mol Ecol Notes..

[34] Toonen RJ, Hughes S (2001). Increased throughput for fragment analysis on ABI Prism 377 automated sequencer using a membrane comb and STRand software. Biotechniques.

[35] van Oosterhout C, Weetman D, Hutchinson WF (2006). Estimation and adjustment of microsatellite null alleles in nonequilibrium populations. Mol Ecol Notes.

[36] Foll M, Gaggiotti OE (2008). A Genome-Scan method to identify selected loci appropriate for both dominant and codominant markers: a Bayesian perspective. Genetics.

[37] Raymond M, Rousset F (1995). GENEPOP (version 1.2): population genetics software for exact tests and ecumenicism. J Hered.

[38] Weir BS, Cockerham CC (1984). Estimating F-statistics for the analysis of population structure. Evolution.

[39] Goudet J. FSTAT, a program to estimate and test gene diversities and fixation indices (version 2.9.3.2). 2001. http://www2.unil.ch/popgen/softwares/fstat.htm.

[40] Wang J, Brekke P, Huchard E, Knapp LA, Cowlishaw G (2010). Estimation of parameters of inbreeding and genetic drift in populations with overlapping generations. Evolution.

[41] Sokal RR, Rohlf JF (1995). Biometry.

[42] Amos W, Worthington Wilmer J, Fullard K, Burg TM, Croxall JP, Bloch D (2001). The influence of parental relatedness on reproductive success. Proc R Soc Lond Biol..

[43] Cubillos LA, Arcos D, Bucarey DA, Canales MT (2001). Seasonal growth of small pelagic fish off Talcahuano, Chile (37°S–73°W): a consequence of their reproductive strategy to seasonal upwelling?. Aquat Living Resour.

[44] Bailey N, Howard FG, Chapman CJ (1986). Clyde *Nephrops*: biology and Fisheries. Proc R Soc Edinb B..

[45] Waldman B, McKinnon JS, Tornhill NW (1993). Inbreeding and outbreeding depression in fishes, amphibians and reptiles. The natural history of inbreeding and outbreeding: theoretical and empirical perspectives.

[46] Fenster CB, Galloway LF (2000). Inbreeding and outbreeding depression in natural populations of *Chamaecrista fasciculata* (Fabacae). Conserv Biol.

[47] Olano-Marin J, Mueller JC, Kempenaers B (2011). Heterozygosity and survival in blue tits (*Cyanistes caeruleus*): contrasting effects of presumably functional and neutral loci. Mol Ecol.

[48] Jourdan-Pineau H, Folly J, Crochet PA, David P (2012). Testing the influence of family structure and outbreeding depression on Heterozygosity-Fitness Correlations in small populations. Evolution.

[49] Acevedo-Whitehouse K, Spraker TR, Lyons E, Melin SR, Gulland F, Delong RL (2006). Contrasting effects of heterozygosity on survival and hookworm resistance in California sea lion pups. Mol Ecol.

[50] Mills LS, Allendorf FW (1996). The one-migrant-per-generation rule in conservation and management. Conserv Biol.

[51] Mytilineou C, Castro M, Gancho P, Fourtouni A (1998). Growth studies on Norway lobster, *Nephrops norvegicus* (L.), in different areas of the Mediterranean Sea and the adjacent Atlantic. Sci Mar..

[52] Gkafas AG, Tsigenopoulos C, Magoulas A, Panagiotaki P, Vafidis D, Mamuris Z (2013). Population subdivision of saddled seabream *Oblada melanura* in the Aegean Sea revealed by genetic and morphometric analyses. Aquat Biol..

[53] Petihakis G, Triantafyllou G, Pollani A, Koliou A, Theodorou A (2005). Field data analysis and application of a complex water column biogeochemical model in different areas of a semi-enclosed basin: towards the development of an ecosystem management tool. Mar Environ Res.

[54] Johnson MS, Wernham J (1999). Temporal variation of recruits as a basis of ephemeral genetic heterogeneity in the western rock lobster *Panulirus cygnus*. Mar Biol.

[55] Voultsiadou E (2005). Demosponge distribution in the eastern Mediterranean: a NW–SE gradient. Helgoland Mar Res..

