# Supplementary material for: Heterozygosity fitness correlations and generation interval of the Norway lobster in the Aegean Sea, eastern Mediterranean
Source: J Biol Res (Thessalon). 2019 Nov 8;26:14. doi: 10.1186/s40709-019-0103-0 (PMC6842237; doi:10.1186/s40709-019-0103-0)

**Table S1.** Number of individuals per gender and month.

| Females | JAN (26) | FEB (8) | MAR (33) | APR (49) | MAY (46) | JUN (17) | JUL (20) | AUG (31) | SEP (41) | OCT (61) | NOV (46) | DEC (25) |
| --- | --- | --- | --- | --- | --- | --- | --- | --- | --- | --- | --- | --- |
| Males | JAN (30) | FEB (42) | MAR (46) | APR (38) | MAY (27) | JUN (9) | JUL( 26) | AUG (22) | SEP (22) | OCT (27 | NOV (38) | DEC (34) |

**Figure S1.** Factorial Correspondence Analyses for all individuals. Result suggest a single population.


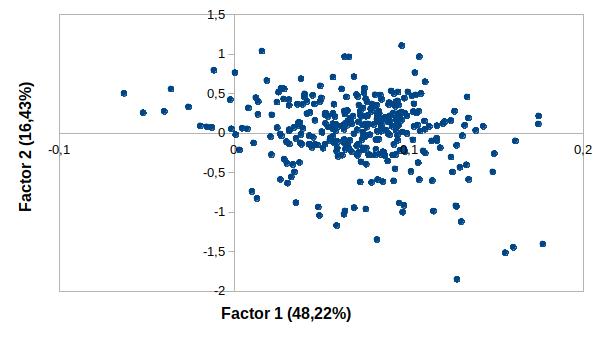

Supplement: Supplementary file 1 — Additional file 1: Table S1. Number of individuals per gender and month. Figure S1. Factorial Correspondence Analyses for all individuals. Result suggest a single population. [file 40709_2019_103_MOESM1_ESM.doc]
